# Marker-Trait Association for Biomass Yield of Potential Bio-fuel Feedstock *Miscanthus sinensis* from Southwest China

**DOI:** 10.3389/fpls.2016.00802

**Published:** 2016-06-07

**Authors:** Gang Nie, Linkai Huang, Xinquan Zhang, Megan Taylor, Yiwei Jiang, Xiaoqing Yu, Xinchun Liu, Xinyu Wang, Yajie Zhang

**Affiliations:** ^1^Department of Grassland Science, Animal Science and Technology College, Sichuan Agricultural UniversityChengdu, China; ^2^Department of Agronomy, Purdue UniversityWest Lafayette, IN, USA; ^3^Department of Agronomy, Iowa State UniversityAmes, IA, USA; ^4^Department of Plant Genetics and Breeding, Agricultural College, Sichuan Agricultural UniversityChengdu, China

**Keywords:** bio-fuel, biomass yield, molecular markers, association analysis, *Miscanthus sinensis*

## Abstract

As a great potential bio-fuel feedstock, the genus *Miscanthus* has been widely studied around the world, especially *Miscanthus* × *giganteus* owing to its high biomass yield in Europe and North America. However, the narrow genetic basis and sterile characteristics of *M*. × *giganteus* have become a limitation for utilization and adaptation to extreme climate conditions. In this study, we focused on one of the progenitors of *M*. × *giganteus, Miscanthus sinensis*, which was originally distributed in East Asia with abundant genetic resources and comparable biomass yield potential to *M*. × *giganteus* in some areas. A collection of 138 individuals was selected for conducting a 3-year trial of biomass production and analyzed by using 104 pairs of SRAP, ISAP, and SSR primers for genetic diversity as well as marker-trait association. Significant differences in biomass yield and related traits were observed among individuals. Tiller number, fresh biomass yield per plant and dry biomass yield per plant had a high level of phenotypic variation among individuals and the coefficient of variation were all above 40% in 2011, 2012, and 2013. The majority of the traits had a significant correlation with the biomass yield except for the length and width of flag leaves. Plant height was a highly stable trait correlated with biomass yield. A total of 1059 discernible loci were detected by markers across individuals. The population structure (Q) and cluster analyses identified three subpopulations in the collection and family relative kinship (K) represented high gene flow among *M. sinensis* populations from Southwest China. Model testing identified that Q+K was the best model for describing the associations between the markers and traits, compared to the simple linear, Q or K model. Using the Q+K model, 12 significant associations (*P* < 0.001) were identified including four markers with plant height and one with biomass yield. Such associations would serve an efficient tool for an early selection of *M. sinensis* and facilitate a genetic improvement of biomass yield for this species.

## Introduction

The growing use of fossil fuel has contributed to increasing global warming, but the uses of renewable energy resources such as bio-fuels could be an efficient approach to solve the energy challenge (Kim et al., 2014). The genus *Miscanthus*, comprising of C4 perennial warm-season rhizomatous grasses (Lewandowski et al., 2003b), is a promising non-food bio-energy crop for cellulosic bio-fuel production due to its broad adaptation, potential high biomass productivity, low-nutrient input, and the ability to sequester carbon (Lewandowski et al., 2000; Clifton-Brown et al., 2001; Stewart et al., 2009; Dwiyanti et al., 2014; Anzoua et al., 2015). *Miscanthus* × *giganteus* is a hybrid generated from a cross between tetraploid *Miscanthus sacchariflorus* and diploid *Miscanthus sinensis*. It has been considered as a candidate for bio-fuel production within the genus.

It is generally known that biomass yield is a critical trait for potential bio-energy crops. Extensive research works of biomass yield in *Miscanthus* have been completed in Europe and North America (Greef et al., 1997; Hodkinson et al., 2002a,b; Heaton et al., 2008, 2009; Hastings et al., 2009). *M*. × *giganteus* performs well on biomass yield and is the only hybrid genotype currently available for use in most countries (Nishiwaki et al., 2011; Dwiyanti et al., 2014), but it is time and labor consuming to propagate the plants through rhizome division or tissue culture. Furthermore, it is highly risky and genetically difficult to improve *M*. × *giganteus* through breeding due to the narrow genetic basis and triploid nature of this species, posing limitations to its biomass productivity, climatic adaptation and overwintering survival under some extreme conditions (Lewandowski et al., 2003a; Clark et al., 2014; Anzoua et al., 2015). As a progenitor of *M*. × *giganteus*, diploid *M. sinensis* is a kind of cross-pollination plant which can be propagated by seeds and potentially provides a comparable biomass yield to that of *M*. × *giganteus* in some areas (Zhao et al., 2013; Anzoua et al., 2015; Gifford et al., 2015). Originally distributed in East Asia throughout China, Korea, and Japan, collection of *M. sinensis* has been made and utilized by many research groups for phenotypic characterization and genetic evaluation (Xu et al., 2013; Nie et al., 2014; Yook et al., 2014; Anzoua et al., 2015). Nevertheless, further works for evaluation of domestication and improvement of *M. sinensis* as a new valuable genetic resource need to be conducted, especially in areas of its origin (Yook et al., 2014).

Because *Miscanthus* requires a lengthy establishment phase and there are some challenges in collecting phenotypic data for a large number of individuals, development of genetic markers associated with a trait of interest would be an efficient approach to enhance *Miscanthus* breeding programs (Clifton-Brown and Lewandowski, 2000; Gifford et al., 2015). Prior to the development of a marker-assisted selection program, quantitative trait locus (QTL) mapping using a population derived from a biparental cross would have been performed to establish associations between traits and genetic markers. However, the process of constructing a mapping population for QTL analysis can be lengthy, especially for perennial grasses.

Association mapping, also known as linkage disequilibrium (LD) mapping, has been proved to be useful and powerful for genetic dissection of complex traits (Yu et al., 2011). Compared to linkage mapping in traditional biparental populations, association mapping results in higher mapping resolution and evaluates a wide range of alleles rapidly (Yu and Buckler, 2006). This technique has been successfully applied for investigating some important agronomic traits in model plant and crop species (Aranzana et al., 2005; Breseghello and Sorrells, 2006; Skøt et al., 2007; Eleuch et al., 2008; Harjes et al., 2008; Wang et al., 2008). There were only a few reports on *Miscanthus* association mapping (Zhao et al., 2013; Slavov et al., 2014); meanwhile, QTL studies were conducted on limited genetic maps and population (Atienza et al., 2003a,b,c,d; Gifford et al., 2015; Liu et al., 2015).

The unavailable genome sequence and lack of reliable molecular markers limit *Miscanthus* genetic research. However, the *Miscanthus* genus belongs to the Tribe Andropogoneae (Poaceae) which contains many important C4 crops including maize (*Zea mays* L.), sorghum (*Sorghum bicolor* L. Moench), and sugarcane (*Saccharum officinarum* L.) with rich genomic databases, and a large number of SSRs have been proven to have high transferability to *M. sinensis* (Hernandez et al., 2001; Lu et al., 2012; Xu et al., 2013; Zhao et al., 2013; Chae et al., 2014; Yook et al., 2014). In addition, new PCR-based markers can be developed for amplifying different regions of DNA segment targets without needing prior knowledge of target sequences and they can be used for studying *M. sinensis* genetic diversity, QTL and association mapping.

Southwest China is the major distribution area or diversity center for *M. sinensis*. As one of the new leading candidates to meet biomass demand for future power generation and bio-fuels production, *M. sinensis* needs further genetic improvement using both conventional breeding and modern biotechnical approaches. In previous studies, we used different molecular markers and chloroplast DNA (trnL-F and rpl20-rps12) sequence to detect the genetic diversity and differentiate the collected *M. sinensis* population from southwest China (Xu et al., 2013; Nie et al., 2014; Yan et al., 2015). Although, different population size was used in these studies before, the similar results demonstrated that the population had high gene flow and fairly weak genetic differentiation, which would increase power to detect marker-trait associations. Building on previous studies, we extended the number of PCR-based markers by using simple sequence repeats (SSRs) developed from *M. sinensis* (Hung et al., 2009; Ho et al., 2011; Zhou et al., 2011), maize (Zhong et al., 2009; Lu et al., 2012), sorghum (Wang et al., 2005; Xu et al., 2013), sugarcane (Lu et al., 2012), and SSR developed from conserved expressed sequence tags (ESTs) databases on grass species (Kantety et al., 2002), as well as intron splice position amplified markers of intron sequence amplified polymorphism (ISAP) and parts of sequence related amplified polymorphism (SRAP) markers used in Nie et al. (2014) on 138 diverse *M. sinensis* varieties selected from previous population according to the geographic information (Xu et al., 2013; Nie et al., 2014). We also conducted a 3-year replicated field trail for phenotypic evaluation of the population and combined with genotype data for marker-trait association analysis to identify key loci associated with phenotypic traits related to biomass yield. The research results would be useful for Miscanthus breeding aimed at improvement of biomass and related traits.

## Materials and methods

### Plant material collection and DNA extraction

A total of 138 *M. sinensis* individuals used in this study were selected from previous studies (Xu et al., 2013; Nie et al., 2014) collected from Sichuan, Chongqing, Guizhou, and Yunnan provinces, located in Southwest China. The individual geographic information were listed in Table 1 (The distribution map could see Nie et al., 2014, **Figure 3**). Briefly, each of the genotypes was cloned to three individuals using rhizome division and planted following a complete randomized block design, with one replicate per genotype in each of three blocks. Prior to transplanting, plant leaves were cut back to 8–10 cm with 6–10 tillers. All the individuals were transplanted to the Sichuan Agriculture University farm (Ya' an, Sichuan, China; *N 30*°*08', E 103*°*14'*) in May of 2010, with an average annual precipitation of 1774 mm. The soil pH at the experimental site ranged from 5.3 to 5.5, and soil type was purplish loam with 1.46% organic qualitative content. Plants were well watered immediately after transplanting and no fertilizer or water was applied to the plants afterwards.

**Table 1:** **Geographic information for 138 ***M. sinensis*** individuals in this study**.

**Identity**	**Source**	**Habital**	**Altitude (m)**	**Latitude (N)**
1	Ya'an	Hillside	623.3	29°58′40.2″
2	Laoban Mountain	Forest	633.8	29°58′51.5″
3	Laoban Mountain	Forest	650.7	29°58′42.2″
4	Laoban Mountain	Forest	644.1	29°58′39.3″
5	Laoban Mountain	Fores	644.1	29°58′39.3″
6	Ya'an	Orangery	657.8	29°58′39.1″
7	Bi Feng Xia	Bamboo grove	681.1	30°04′28.5″
8	Bi Feng Xia	Forest	1018	30°04′49.0″
9	Bi Feng Xia	Forest	1018	30°04′49.0″
10	Bi Feng Xia	Hillside	989	30°05′01.9″
11	Bi Feng Xia	Hillside	989	30°05′01.9″
12	Bi Feng Xia	Hillside	904.6	30°05′18.0″
13	Bao Xing	Riverside	1253	30°28′51.0″
14	Bao Xing	Riverside	1253	30°28′51.0″
15	Bao Xing	Riverside	1253	30°28′51.0″
16	Bao Xing	River Valley	1179	32°21′11.3″
17	Bao Xing	River Valley	1179	32°21′11.3″
18	Bao Xing	River Valley	1179	32°21′11.3″
19	Bao Xing	Highway side	924	30°20′32.7″
20	Erlang Mountain	Forest	2091	29°52′55.0″
21	Erlang Mountain	Forest	2091	29°52′55.0″
22	Erlang Mountain	Hillside	1650	29°53′25.0″
23	Erlang Mountain	Hillside	1650	29°53′25.0″
24	Erlang Mountain	Hillside	1605	29°53′44.5″
25	Erlang Mountain	Hillside	1605	29°53′44.5″
26	Erlang Mountain	Hillside	1419	29°56′43.1″
27	Tuowu Mountain	Forest	1630	29°02′41.52″
28	Tuowu Mountain	Forest	1630	29°02′41.52″
29	Tuowu Mountain	Forest	1630	29°02′41.52″
30	Tuowu Mountain	Forest	1630	29°02′41.52″
31	Tuowu Mountain	Forest	1630	29°02′41.52″
32	Niba Mountain	Forest	1626	29°42′47.5″
33	Niba Mountain	Forest	1626	29°42′47.5″
34	Niba Mountain	Hillside	1594	29°43′07.7″
35	Niba Mountain	Hillside	1594	29°43′07.7″
36	Renshou	Highway	432.2	30°00′26.6″
37	Renshou	Highway	432.2	30°00′26.6″
38	Renshou	Highway	432.9	30°00′06.6″
39	Renshou	Highway	432.9	30°00′06.6″
40	Renshou	Bushes	471.8	30°00′16.6″
41	Renshou	Bushes	471.8	30°00′16.6″
42	Hongya	Bushes	483.5	29°53′22.6″
43	Hongya	Bushes	520.8	29°49′48.7″
44	Hongya	Dam side slope	487.4	29°50′26.8″
45	Hongya	Dam side slope	493.1	29°50′26.6″
46	Hongya	Dam side slope	501	29°53′12.6″
47	Zizhong	Orangery	350.1	29°49′03.4″
48	Zizhong	Orangery	350.1	29°49′03.4″
49	Zizhong	Orangery	350.1	29°49′03.4″
50	Zizhong	Orangery	350.1	29°49′03.4″
51	Zizhong	Bushes	348.2	29°49′06.0″
52	Luzhou	Rice ridge	318.7	28°52′44.9″
53	Luzhou	Rice ridge	318.7	28°52′44.9″
54	Luzhou	Rice ridge	318.7	28°52′44.9″
55	Luzhou	Rice ridge	321	28°52′42.7″
56	Luzhou	Bushes	241.6	28°49′01.7″
57	Luzhou	Bushes	241.6	28°49′01.7″
58	Luzhou	Bushes	241.6	28°49′01.7″
59	Luzhou	Bushes	241.6	28°49′01.7″
60	Yibin	Cityside	500	28°45′08.8″
61	Yibin	Cityside	500	28°45′08.8″
62	Yibin	Cityside	500	28°45′08.8″
63	Yibin	Riverside	317.5	28°45′31.0″
64	Yibin	Riverside	342.4	28°50′42.0″
65	Zigong	Grass Bushes	353.5	29°26′37.4″
66	Zigong	Grass Bushes	353.5	29°26′37.4″
67	Jiangyou	Roadside	564.4	31°56′59.5″
68	Jiangyou	Roadside	564.4	31°56′59.5″
69	Jiangyou	Roadside	564.4	31°56′59.5″
70	Jiangyou	Highway	616.8	31°59′45.6″
71	Jiangyou	Highway	616.8	31°59′45.6″
72	Jiangyou	Highway	641.2	32°03′04.4″
73	Jiangyou	Highway	641.2	32°03′04.4″
74	Jiangyou	Highway	641.2	32°03′04.4″
75	Jiangyou	Highway	687.8	32°04′26.4″
76	Jiangyou	Highway	687.8	32°04′26.4″
77	Jian'ge	Hillside	611	32°13′58.3″
78	Jian'ge	Hillside	611	32°13′58.3″
79	Guangyuan	Hillside	612.1	32°38′16.9″
80	Guangyuan	Hillside	612.1	32°38′16.9″
81	Guangyuan	Hillside	612.1	32°38′16.9″
82	Guangyuan	Hillside	612.1	32°38′16.9″
83	Guangyuan	Hillside	612.1	32°38′16.9″
84	Guangyuan	Hillside	612.1	32°38′16.9″
85	Guangyuan	Bushes	644.8	32°38′57.9″
86	Guangyuan	Bushes	644.8	32°38′57.9″
87	Guangyuan	Bushes	644.8	32°38′57.9″
88	Daying	Highway	327.8	30°36′36.7″
89	Daying	Highway	327.8	30°36′36.7″
90	Daying	Highway	327.8	30°36′36.7″
91	Daying	Highway	327.8	30°36′36.7″
92	Daying	Highway	327.8	30°36′36.7″
93	Daying	Highway	327.8	30°36′36.7″
94	Shapingba	Riverside	255.3	29°39′39.9″
95	Shapingba	Riverside	255.3	29°39′39.9″
96	Banan	Hillside	476.5	29°31′10.7″
97	Banan	Hillside	476.5	29°31′10.7″
98	Banan	Forest edge	476.5	29°31′10.7″
99	Banan	Forest edgean	476.5	29°31′10.7″
100	Banan	Forest edge	476.5	29°31′10.7″
101	Banan	Forest edge	476.5	29°31′10.7″
102	Banan	Forest edge	476.5	29°31′10.7″
103	Banan	Forest edge	476.5	29°31′10.7″
104	Nanchuan	Bushes	579.4	29°09′25.9″
105	Nanchuan	Bushes	579.4	29°09′25.9″
106	Nanchuan	Bushes	579.4	29°09′25.9″
107	Nanchuan	Hillside	579.4	29°09′25.9″
108	Nanchuan	Hillside	579.4	29°09′25.9″
109	Nanchuan	Hillside	579.4	29°09′25.9″
110	Nanchuan	Hillside	579.4	29°09′25.9″
111	Dabai	Hill foot	455.9	28°29′26.1″
112	Dabai	Hill foot	455.9	28°29′26.1″
113	Dabai	Hill foot	455.9	28°29′26.1″
114	Dabai	Hill foot	455.9	28°29′26.1″
115	Zunyi	Conifer forest	914.7	27°46′18.8″
116	Zunyi	Conifer forest	914.7	27°46′18.8″
117	Zunyi	Conifer forest	914.7	27°46′18.8″
118	Zunyi	Conifer forest	914.7	27°46′18.8″
119	Zunyi	Conifer forest	914.7	27°46′18.8″
120	Zunyi	Conifer forest	914.7	27°46′18.8″
121	Guiyang	Field ridge	1286	27°42′52.1″
122	Guiyang	Field ridge	1286	27°42′52.1″
123	Guiyang	Field ridge	1286	27°42′52.1″
124	Guiyang	Field ridge	1287	26°42′20.1″
125	Guiyang	Dam side slope	1268	26°30′20.2″
126	Guiyang	Dam side slope	1268	26°30′20.2″
127	Guiyang	Dam side slope	1268	26°30′20.2″
128	Zhenning	Hillside	1284	26°02′35.6″
129	Zhenning	Hillside	1284	26°02′35.6″
130	Zhenning	Hillside	1284	26°02′35.6″
131	Zhenning	Hillside	1284	26°02′35.6″
132	Huangguoshu	Forest edge	946.5	25°58′22.5″
133	Huangguoshu	Forest edge	946.5	25°58′22.5″
134	Huangguoshu	Forest edge	946.5	25°58′22.5″
135	Huangguoshu	Forest edge	946.5	25°58′22.5″
136	Huangguoshu	Forest edge	946.5	25°58′22.5″
137	Yuxi	Bushes	1721	24°12′15.9″
138	Yuxi	Bushes	1721	24°12′15.9″

Fresh young leaves from each individual were collected for genomic DNA extraction using the Plant Genomic DNA kit (Tiangen®, China) according to the manufacturer's protocol. The quality and concentration of the DNA were determined by comparing the sample with known standards of lambda DNA on 0.8% (w/v) agarose gels and NanoDrop ND-1000 spectrophotometer (NanoDrop Technologies Inc., Rockland, DE, USA). The isolated genomic DNA was diluted to 20 ng/μL for PCR amplification.

### Primer selection and PCR amplification

In this study, we selected part of SRAP primers published previously (Li and Quiros, 2001; Nie et al., 2014) to conduct the association analysis. In addition, six individuals of *M. sinensis* that varied in morphology and geographic locations were selected for screening other markers based on Nie et al. (2014), including 72 ISAP primer combinations (Lu et al., 2008) and 117 SSR primer combinations (Kantety et al., 2002; Wang et al., 2005; Hung et al., 2009; Zhong et al., 2009; Ho et al., 2011; Zhou et al., 2011; Lu et al., 2012). All the primers used in this study were synthesized by Nanjing GenScript Biological Technology & Service (China).

For PCR amplification, the total volume of each PCR reaction system was 20 μl, containing 3 μL template DNA (20 ng/μL), 10 μL of Mix (10 × reaction buffer, 2.0 mM Mg^2+^, 0.6 mM of each dNTP, Tiangen®, China), 0.8 μL primers (10 pmol/μL), 0.4 μL Golden DNA Polymerase (2.5 U/μL, Tiangen®, China) and 5 μL of ddH_2_O. Amplification was performed on a Peltier Thermal Cycler (DNA Engine®, Bio-Rad, USA) under the following conditions: for SRAP and ISAP amplification, 5 min at 94°C for 1 cycle, followed by 5 cycles at 94°C for 1 min, 35°C for 1 min, and 72°C for 1 min, and then 35 cycles at 94°C for 1 min, 50°C for 1 min, and 72°C for 1 min, extended at 72°C for 10 min, then stored at 4°C; for SSR, 5 min at 94°C for 1 cycle, followed by 10 cycles at 94°C for 1 min, 55°C for 30 s, and 72°C for 40 s, decreased 0.5°C for annealing with each cycle, and then 35 cycles at 94°C for 1 min, 50°C for 30 s, and 72°C for 40 s, extended at 72°C for 10 min, then stored at 4°C. Electrophoresis was performed in a denaturing 6% polyacrylamide gel (acrylamide: bis-acrylamide 19:1, 1 × TBE) to separate allele sizes. The gel was stained by AgNO_3_ solutions.

### Phenotypic data collection and analysis

Three morphological traits were measured at early harvest season in 2011. Plant height (H) was measured at the ground level to the top of the plant. The total number of tillers in each plant (TN) was counted after harvest. The fresh biomass yield of per plant (fresh weight, FW) was evaluated with autumn harvest in October. In 2012 and 2013, in addition to H, TN, and FW, several other morphological traits associated with biomass were measured. The main tiller diameter (TD) was measured approximately 10–15 cm from the base of the plant on three randomly chosen tillers. Number of main stem internodes (NI) was counted and the length of the main internode (LI) was measured. The length of flag leaf (LF) and length of longest leaf (LL) were measured from the ligule to the tip along the central vein of the leaf. The width of flag leaf (WF) and width of longest leaf (WL) were measured for the width of the blade at half-leaf length for the leaf which was recorded for measuring the length. Plants were harvested about 20 cm above the soil surface, and the whole above-ground biomass was weighed as FW in October. The harvested tissue were then dried in an oven at 105°C for 1 h, followed by 70°C for 3 days, for determining dry biomass yield per plant (dry weight, DW). All the plants in the field were cut about 20 cm to avoid rhizome damage and facilitate quick re-growth in the following season.

Analysis of variance (ANOVA) and correlation analysis of morphological traits were performed using SPSS 17.0 software (IBM, Armonk, New York, USA). Effects of both environment (different measurement year) and individuals on various traits were determined using the Least Significant Difference test model. Pearson correlation coefficients were calculated for correlation analysis. The coefficient of variation (CV) was calculated using the following model—CV = SD/Mean ^*^ 100%—for detecting the discrete level of the data.

### Genetic diversity and population structure

The alleles of molecular markers were scored manually for the population as band presence (1) or absence (0), and each of them was treated as an independent character regardless of its intensity. A present/absent data matrix was constructed to analyze the genetic diversity and population structure. The discriminatory power of different primers was evaluated by means of polymorphic information content (*PIC*), calculated by the following model *PIC*_*i*_ = 2*f*_*i*_(1 − *f*_*i*_) (Roldan-Ruiz et al., 2000). In the model, *PIC*_*i*_ is the polymorphic information content of marker “*I*,” *fi* is the frequency of the amplified allele (band present), and 1 −*f*_*i*_ is the frequency of the null allele.

Population structure (Q) of 138 *M. sinensis* individuals was confirmed using the model-based clustering approach implemented in STRUCTURE v2.3.4 software (Pritchard et al., 2000) with the “admixture model,” burn-in period of 100,000 iterations and a run of 100,000 replications of Markov Chain Monte Carlo (MCMC) after burn in. For each run, 20 independent runs of STRUCTURE were performed with the number of clusters (K) varying from 1 to 10. Maximum likelihood and delta K (ΔK) tests were used to determine the optimum number of subgroups (Evanno et al., 2005). For clustering analysis, the similarity coefficients were used to construct an unweighted pair group method with arithmetic means (UPGMA) dendogram using sequential agglomerative hierarchical and nested clustering (SAHN) module in the NTSYS-pc version2.10 software. Analysis of molecular variance (AMOVA) was used to calculate variation among and within populations using GenAlEx ver. 6.41 (Peakall and Smouse, 2012).

All genetic diversity indices were calculated using PopGen32 v.1.31, assuming Hardy-Weinberg equilibrium; the genetic diversity was evaluated with parameters: Nei's (1973) gene diversity (H) and Shannon's Information Index of Diversity (I). The total gene diversity (H_T_) was divided into gene diversity within populations (H_S_) and the gene diversity among populations (D_ST_). These parameters were calculated according to the equation *H*_*T*_ = *H*_*S*_+*D*_*ST*_. The genetic differentiation coefficient (G_ST_) was calculated as a ratio of D_ST_/H_T_ and was used to measure population differentiation. Gene flow was calculated as *N*_*m*_ = 0.5(1 − *G*_*ST*_)∕*G*_*ST*_ to estimate the level of gene drift among the populations (Slatkin and Barton, 1989).

### Marker-trait association analysis

The markers with minor allele frequency less than 5% were removed in order to reduce false positive associations. Relative kinship (K) among samples was calculated by TASSEL 2.1 software. The marker-trait association analysis was conducted to reveal associations between the interest traits and marker alleles using TASSEL 2.1 software along with the General Linear Model (GLM) and Mixed Linear Model (MLM) procedure (Bradbury et al., 2007) to control for population structure and relative kinship. The simple linear model, Q (population structure results included as fixed effects generating from STRUCTURE software) model, K (relative kinship results included as fixed effects generating from TASSEL software) model, and Q+K models were tested to identify the best model fitting biomass related traits using Quantile-quantile (QQ) plots for association mapping in the *M. sinensis* populations. Two thresholds for significant associations were tested in our study. First, the significance threshold for associations between loci and traits was set at *P* < 0.001. Second, the Bonferroni correction of multiple testing (*P* < 0.05/934 ~5.35 × 10 −^5^) was performed based on q-value using false discovery rate (FDR, α_*c*_ = 0.05). The phenotypic variation explained by the single associated marker (*R*^2^) indicated the fixed marker effects.

### Genome-wide prediction

The genome-wide prediction was carried out by using the R package rrBLUP (Endelman, 2011) with ridge regression. The average correlation between the predicted phenotypic values from marker data and the original phenotypic values directly from field trail was used as the criteria of genome prediction accuracy. The accuracy (Pearson's correlation coefficient) was calculated with recommended 10-fold cross-validation and was repeated 100 times (Slavov et al., 2014). The adjusted prediction accuracy was calculated by dividing accuracy by the square root of the broad-sense heritability (h^2^), where h^2^ was calculated by using PROC MIXED (SAS Institute, Version 9.1, Cary, NC, USA). The h^2^ was calculated as follows: h^2^ = $\sigma_{\text{g}}^{2}$/($\sigma_{\text{g}}^{2}+\sigma_{\text{e}}^{2}$/re $+\sigma_{\text{ge}}^{2}$/e), where $\sigma_{\text{g}}^{2}$, $\sigma_{\text{e}}^{2}$, $\sigma_{\text{ge}}^{2}$ represent Type III SS (sums of squares) for genotype (G), environment (E), and G × E, respectively. The “e” is the degree of freedom of environment and “re” is the degree of freedom of G × E.

## Results

### Phenotypic variation and correlation

Significant differences among individuals were observed through ANOVA analysis for all measured traits (Table 2). In addition, the biomass yield per plant was increased year by year after establishment. Significant increases were noted in the mean of fresh biomass yield per plant—498.7 g in 2011, 770.8 g in 2012 and 1001.9 g in 2013, with the highest individual increased from 1350 g in 2011 to 2225 g in 2013. The results showed that TN, FW, and DW had a high level of phenotypic variation with CV of above 40% in 2011, 2012, and 2013.

**Table 2:** **The mean, range, standard deviation (SD), coefficient of variation (CV) and ***F***-value of plant height (H), tiller number (TN), fresh biomass yield each plant (FW), dry biomass yield each plant (DW), the main tiller diameter (TD), the number of main stem internodes (NI), the length of main internode (LI), the length of flag leaf (LF), the width of flag leaf (WF), the length of longest leaf (LL), and the width of longest leaf (WL) in ***Miscanthus sinensis*** population in 2011, 2012, and 2013**.

**Trait**	**Year**	**Mean**	**Range**	**SD**	**CV (%)**	***F***
H (cm)	2011	203.67	125.31–330.58	41.46	21.98	4.24^***^
	2012	205.18	120.63–307.14	37.58	18.45	
	2013	188.61	111.00–313.62	30.95	15.08	
TN	2011	29.30	4–73	15.18	51.81	6.61^***^
	2012	38.21	5–138	25.94	67.87	
	2013	43.38	9–92	19.04	43.88	
FW (g)	2011	498.7	50–1350	250.98	50.32	6.08^***^
	2012	770.8	85–2045	444.64	57.69	
	2013	1001.9	200–2225	474.35	47.35	
DW (g)	2012	363.8	55–1170	220.49	60.61	8.923^***^
	2013	399.0	105–875	180.47	45.23	
TD (cm)	2012	0.535	0.277–0.859	0.10	19.62	9.346^***^
	2013	0.579	0.193–0841	0.12	20.67	
NI	2012	10.50	6–16	2.30	21.93	3.952^***^
	2013	12.34	7–17	2.22	17.95	
LI (cm)	2012	9.07	4.32–15.68	2.33	25.74	3.785^***^
	2013	10.97	5.49–20.63	2.47	22.52	
LF(cm)	2012	32.13	7.03–77.53	14.07	43.80	9.626^***^
	2013	35.10	11.33–70.33	11.89	33.88	
WF (cm)	2012	0.87	0.33–1.87	0.30	34.46	6.009^***^
	2013	0.97	0.52–2.43	0.37	38.07	
LL (cm)	2012	77.63	35.21–121.78	14.89	19.19	6.756^***^
	2013	82.36	53.97–119.78	12.47	15.14	
WL (cm)	2012	1.59	0.72–2.47	0.36	22.40	12.213^***^
	2013	1.83	1.10–3.12	0.41	22.53	

*** *Significant differences at P < 0.001*.

Significant positive correlations of biomass yield (both fresh and dry) with TN, H, TD, NI, LI, LL, and WL were found, while no correlations were seen in with LF and WF (Table 3). The higher correlation coefficients indicated that *M. sinensis* biomass yield in the field was largely influenced by TN and H. On the other hand, TN had a significant negative correlation with tiller diameter (*r* = −0.185, *P* < 0.05) and leaf length (*r* = −0.287, *P* < 0.01), indicating that a *M. sinensis* plant with a high number of tillers always followed with small tiller diameter and low leaf length. Plant height had a significant positive correlation with the main internode length (*r* = 0.522, *P* < 0.01). Significant positive correlations were also found between leaf width and tiller diameter and between flag leaf length and flag leaf width.

**Table 3:** **Pearson correlation coefficients among TN, H, FW, TD, NI, LI, LF, WF, LL, WL, and DW^§^ in ***M. sinensis*** population^‡^**.

	**TN**	**H**	**FW**	**TD**	**NI**	**LI**	**LF**	**WF**	**LL**	**WL**	**DW**
TN	1										
H	−0.028	1									
FW	0.610^**^	0.408^**^	1								
TD	−0.185^*^	0.204^*^	0.364^**^	1							
NI	−0.030	0.391^**^	0.285^**^	0.218^*^	1						
LI	−0.011	0.522^**^	0.223^**^	0.002	0.301^**^	1					
LF	−0.125	0.246^**^	0.042	0.095	−0.131	0.062	1				
WF	−0.138	0.283^**^	0.110	0.229^**^	0.117	0.181^*^	0.525^**^	1			
LL	−0.136	0.380^**^	0.264^**^	0.386^**^	0.085	0.212^*^	0.427^**^	0.208^*^	1		
WL	−0.287^**^	0.331^**^	0.285^**^	0.527^**^	0.360^**^	0.195^*^	0.053	0.406^**^	0.360^**^	1	
DW	0.527^**^	0.392^**^	0.897^**^	0.334^**^	0.386^**^	0.251^**^	−0.006	0.131	0.170^*^	0.364^**^	1

§ *TN, tiller number; H, plant height; FW, fresh biomass yield each plant; TD, the main tiller diameter; NI, the number of main stem internode; LI, the length of main internode; LF, the length of flag leaf; WF, the width of flag leaf; LL, the length of longest leaf; WL, the width of longest leaf; and DW, dry biomass yield each plant*.

‡ *Correlation calculated using mean of 3 years*.

* *Correlation is significant at P < 0.05*.

** *Correlation is significant at P < 0.01*.

### Genotypic variation and population structure

A total of 104 pairs of primers (Supplementary Table 1) were screened for genotyping the collections of 138 *M. sinensis* individuals while the other primers failed to amplify or did not produce clear bands. In total, 1059 bands were produced and 993 (93.8%) were polymorphic. For the SSR primers developed from *M. sinensis*, sorghum, sugarcane, maize, and conserved ESTs in grasses, the average of bands produced per primer was 7.8, 8.9, 5.8, 7.2, and 8.0, respectively. The production of ISAP primers had a similar result (6.5) with SSR, while the SRAP had a higher productive capacity (19.8). The mean of polymorphic information content ranged from 26.7% (SSR-4) to 39.0% (SSR-5), demonstrating a different discriminatory capacity for each kind of primer (Table 4).

**Table 4:** **The amplification results of each primer and the comparison of productive capacity among seven primers**.

**Primer**					**PPB**	**PIC**	**Origin developed**
**kind**	**NPC**	**TB**	**TPB**	**ANB**	**(%)**	**(%)**	
SSR-1	25	195	193	7.8	99.0	0.343	*M. sinensis*
SSR-2	7	62	60	8.9	96.8	0.350	*Sorghum*
SSR-3	9	52	45	5.8	86.5	0.282	*Saccharum*
SSR-4	9	65	56	7.2	86.2	0.267	*Zea mays*
SSR-5	10	80	78	8.0	97.5	0.390	*Conserved grass ESTs*
SRAP	24	475	442	19.8	93.1	0.341	\
ISAP	20	130	119	6.5	91.5	0.275	\
Total	104	1059	933	\	\	\	\

*NPC, Number of primer combinations; TB, Total number of bands produced; TPB, Total number of polymorphic bands produced; ANB, The average number of bands produced; PPB (%), Percentage of polymorphic bands; PIC (%), Polymorphic information content*.

**Figure 1:**
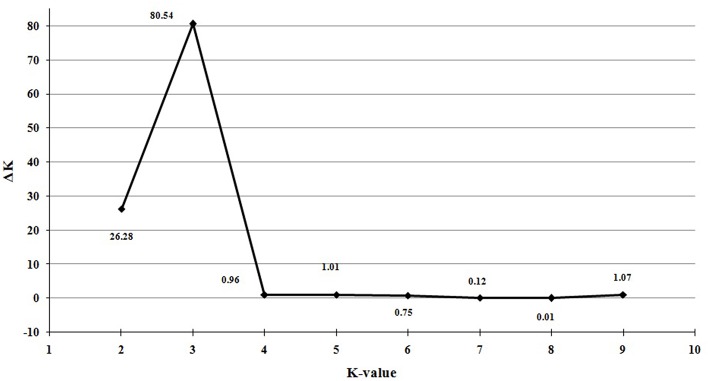
**Optimal value of K determined by delta K (△K)**.

**Figure 2:**
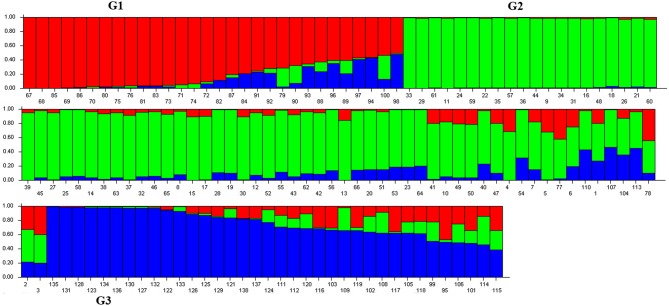
**Population structure analysis of 138 ***M. sinensis individuals from southwest China*****. Numbers on the x-axis indicate the individual and numbers on the y-axis show the group membership. G1, G2, and G3 represent the identified structure groups.

Population structure of the 138 individuals was estimated under the Hardy-Weinberg Equilibrium by using STRUCTURE V2.3.3 software. After dropping the markers with minor allele frequency less than 5%, the total number of marker loci retained for structure and association analysis was 934. Based on maximum likelihood and delta K (ΔK) values, the number of optimum subgroups was three (Figure 1). Accordingly, the 138 individuals were assigned into these three groups. Among them, 34 individuals were assigned to G1, 66 individuals to G2, and 38 individuals to G3 (Figure 2). By using a membership probability threshold (*Q*-value) of 0.60, the majority of the individuals were clearly assigned to the specific groups while admixture between groups referred to 18 individuals with *Q* < 0.6 (data not shown).

The genetic similarities coefficient (GS) values of 138 individuals ranged from 0.59 to 0.95 with an average of 0.67. The UPGMA dendrogram based on GS data obviously revealed three major clusters similar to the result from the population structure analysis when the GS value was equal to 0.67 (Supplementary Figure 1).

The three groups comprised of 138 individuals had a relatively high genetic diversity reflected by Nei's (1973) gene diversity (H) and Shannon's Information Index of Diversity (I) (Table 5). Total gene diversity (H_T_) was 0.35 ± 0.015, while gene diversity within groups (H_S_) was 0.33 ± 0.014 and gene diversity among groups (D_ST_) was 0.016. The total Shannon's Information Index of Diversity (SII) among 138 individuals was 0.52 ± 0.015 with the average of SII within groups was 0.50. The mean genetic differentiation coefficient (G_ST_) was estimated from the 933 bands with a value of 0.046. A higher level of genetic variation within the populations than among them suggested a high frequency of gene flow (*Nm* = 10.32) between the groups. The AMOVA analysis of the *M. sinensis* populations showed similar results, and both the genetic variations within (96.0%) and among (4.0%) groups were significant (*P* < 0.05) (Table 6).

**Table 5:** **Genetic diversity of ***M. sinensis*** populations**.

**Population identity**	**Sample size**	**Na**	**Ne**	**H**	**I**
G1	34	1.97	1.55	0.33	0.50
G2	66	1.99	1.57	0.34	0.51
G3	38	1.98	1.55	0.32	0.49
Mean		1.98	1.56	0.33	0.50
Within Species	138	2.00	1.58	0.35	0.52

*Na, Observed number of alleles averaged across loci; Ne, Effective number of alleles averaged across loci; H, Nei's (1973) gene diversity; I, Shannon's Information index*.

**Table 6:** **AMOVA analysis of ***M. sinensis*** groups**.

**Source of variation**	**Degree of freedom**	**Sum of square**	**Summary of matches**	**Percentage of (%) variation**	***P*-value**
Among Groups	2	897.9	448.9	4%	0.035
Within Groups	135	23235.8	172.1	96%	0.010
Total	137	24133.7		100%	

### Marker-trait association analysis

**Figure 3:**
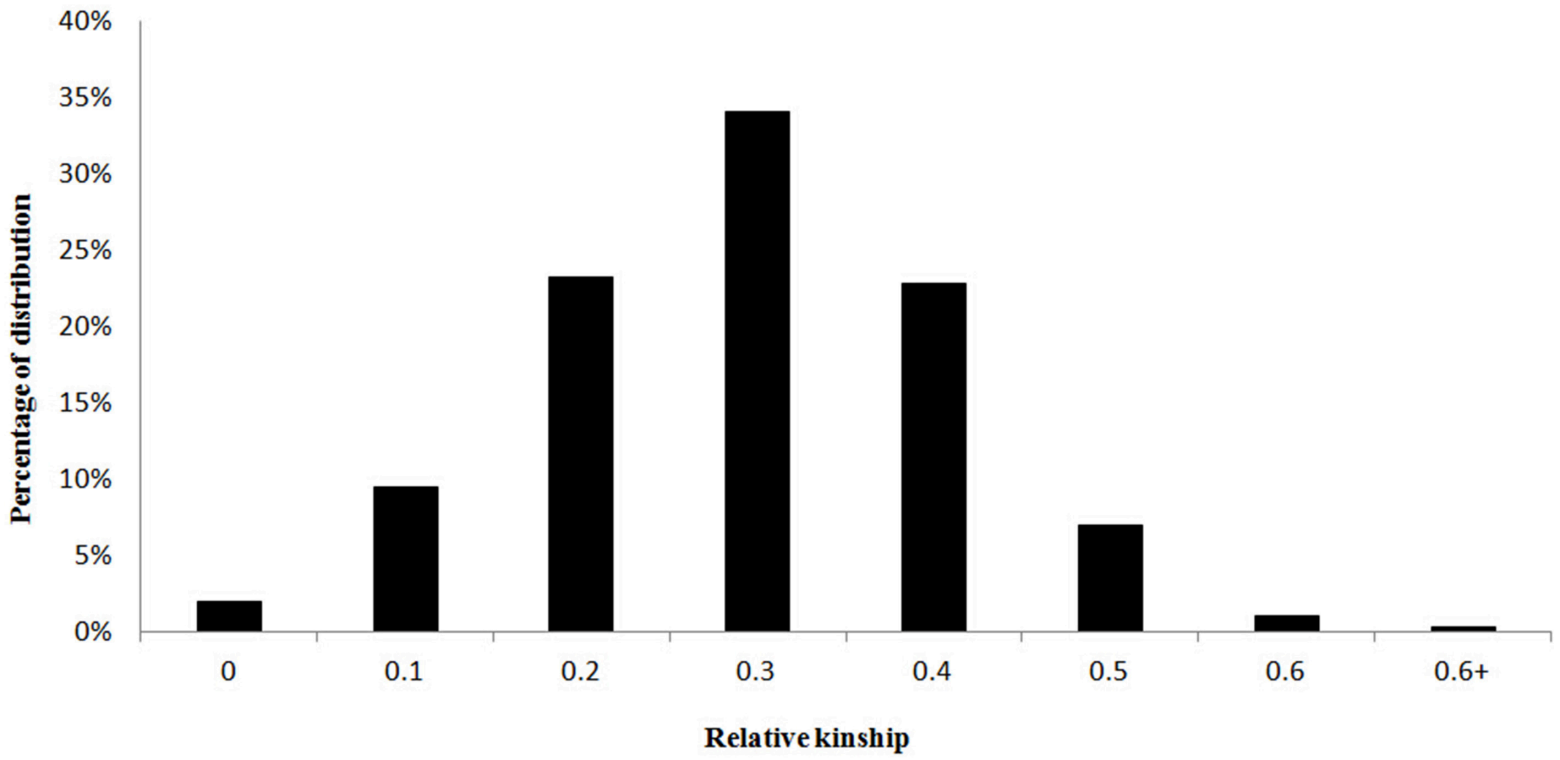
**The distributions of pair-wise kinship coefficients for 138 ***M. sinensis*** individuals**.

**Figure 4:**
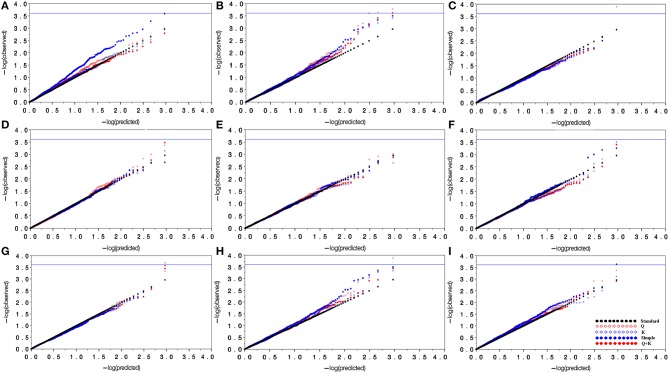
**Quantile-quantile plots for model comparison with biomass yield and related traits**. **(A)**, Evaluation of model types using markers for tiller number; **(B)**, Evaluation of model types using markers for plant height; **(C)**, Evaluation of model types using markers for tiller diameter; **(D)**, Evaluation of model types using markers for internode number; **(E)**, Evaluation of model types using markers for internode length; **(F)**, Evaluation of model types using markers for leaf length; **(G)**, Evaluation of model types using markers for leaf width; **(H)**, Evaluation of model types using markers for fresh biomass yield; **(I)**, Evaluation of model types using markers for dry biomass yield. In this figure, black dots line represent the predicted value equal to the observed value; blue dots represent the simple linear model (without population structure and relative kinship); red diamond represents the Q model; blue diamond represents the K model; and red dots represent the Q+K model.

Marker-based relative kinship estimates have proven useful for quantitative inheritance studies in different populations. For the 138 *M. sinensis* individuals, the pair-wise relative kinship (K) estimates represented a normal distribution with approximately 98% of individuals from 0 to 0.5 (Figure 3). The results agreed that a high gene flow existed among samples. Quantile-quantile (QQ) plot is a probability plot, which is a graphical method of comparing two probability distributions (observed vs. expected). In this study, Q and K were detected among samples. Therefore, the association analysis was performed by taking Q and K into account using GLM and MLM approaches in the software TASSEL 2.1. Biomass yield and related traits were used to test the model with Q only matrix, K only matrix, Q+K matrix and simple linear model (S) excluding the Q and K in QQ plots (Figure 4).

In most cases, the Q+K model and the K model had similar power and demonstrated the best approximation to the excepted cumulative distribution of *P*-values, followed by the Q and S model. The results from the Q+K and K models showed a significant improvement in goodness of fit compared with the other models, except that the fresh biomass and dry biomass yields in the Q+K model had a slightly higher power than the K model. At last, the Q+K model was selected as the best fitting model for association analysis.

A total of 21 significant associations were detected using a simple linear model, 18 using Q model, 15 using K model and 12 using Q+K model (*P* < 0.001). The averages of the phenotypic variations explained by the model for significant associations were 9.2% (S), 10.9% (Q), 46.8% (K), and 47.1% (Q+K), which was consistent with the model test results. For the significant associations detected by Q+K model, 4 markers were associated with plant height, 3 markers with flag leaf width, 1 marker with internode number, and 1 marker with fresh biomass yield (Table 7). In addition, marker “494” was associated with tiller diameter, leaf length and leaf width simultaneously. When comparing the significant associations detected by the Q+K model and the K model, 3 biomass yields related associations were filtered out (*P* > 0.001) in the Q+K model while they significant in the K model. The results were consistent with the model test above that the Q+K and K models had nearly the same capacity to detect the associations but the Q+K model seems a little better fit for biomass to control false positive associations. Specifically, one of associations (marker “793” for flag leaf width) reached genome-wide significant after Bonferroni correction for multiple testing (*P* < 0.05/934 ~ 5.35 × 10^−5^), with an estimated false discovery rate (FDR) < 0.05.

**Table 7:** **Significant marker-trait association of ***M. sinensis*** individuals**.

**Trait**	**Allele number**	**Primer number**	**Locus**	**Allele size (bp)**	***R*^2^ (%)**
Plant Height	86	N-13	ISAP-F6+R7	156	5.39^***^
Plant Height	737	N-89	SRAP-Me1+em8	250	4.43^***^
Plant Height	859	N-95	SRAP-Me10+em2	343	4.55^***^
Plant Height	945	N-98	SRAP-Me6+em10	137	5.06^***^
Tiller Diameter	494	N-70	SSR-SG26	127	6.79^***^
Leaf Length	494	N-70	SSR-SG26	127	5.36^***^
Leaf Width	494	N-70	SSR-SG26	127	5.80^***^
Internode Number	334	N-46	SSR-HAU-12	78	5.48^***^
Flag Leaf Width	14	N-2	ISAP-F1+R3	127	4.94^***^
Flag Leaf Width	335	N-47	SSR-HAU-58	375	5.11^***^
Flag Leaf Width	793	N-92	SRAP-Me6+em8	232	8.43^***^
Fresh Biomass yield	927	N-98	SRAP-Me6+em10	380	5.17^***^

*** *Significant association at P < 0.001 with FDR correction at α_c_ = 0.05; R^2^, Phenotypic variation explained by markers*.

For an overall measure of quality of the genotype and phenotype data, genome-wide prediction was conducted in this study (Table 8). Most of measured traits were moderately heritable with the total average of broad-sense heritability (h^2^) equal to 0.56. Furthermore, the average of prediction accuracy and adjust prediction accuracy was 0.24 and 0.33, respectively. Although, the values of prediction accuracy seem lower, the adjust accuracy of genome-wide prediction for flag leaf width had moderate predictive ability (0.59). Interestingly, association analysis also showed that one marker was highly associated with flag leaf width on genome-wide significant level.

**Table 8:** **Performance of genome-wide prediction in 138 ***Miscanthus sinensis*** genotypes based on 934 markers**.

**Trait**	**Heritability**	**Accu^b^**	**SD^c^_Accu_**	**Adjusted Accu^d^**	**SD^e^_Ad Accu_**
Plant Height	0.67	0.20	0.046	0.24	0.056
Tiller Number	0.74	0.23	0.033	0.27	0.039
Fresh Biomass yield	0.69	0.11	0.046	0.13	0.056
Dry Biomass yield	0.62	0.23	0.035	0.29	0.044
Tiller Diameter	0.62	0.10	0.039	0.13	0.050
Internode Number	0.33	0.20	0.040	0.35	0.069
Internode Length	0.42	0.28	0.031	0.44	0.048
Flag Leaf Length	0.46	0.29	0.030	0.43	0.045
Flag Leaf Width	0.47	0.41	0.024	0.59	0.035
Leaf Length	0.49	0.23	0.031	0.33	0.044
Leaf Width	0.63	0.31	0.030	0.39	0.038
Total Average^a^	0.56	0.24	0.035	0.33	0.048

a *Total Average, overall average and standard deviation across traits*.

b *Accu, average predicted accuracy across 100 random 10-fold cross-validations based on 934 markers*.

c *SD_Accu_, averagestandard deviation of predicted accuracy*.

d *Adjusted Accu, average adjusted predicted accuracy of genome-wide prediction across 100 random 10-fold cross-validations based on 934 markers*.

e *SD_AdAccu_, average standard deviation of adjusted predicted accuracy*.

## Discussion

*Miscanthus* is a typical perennial grass species that requires a long period of time for establishment after transplanting clonal replicates prior to reaching the maximum growth for optimum and stable productivity (Clifton-Brown and Lewandowski, 2000; Anzoua et al., 2015). *M. sinensis* grows slowly at the initial phase of establishment due to uneven splitting of the rhizome, the differences in growing conditions prior to transplanting, and variable adaptive capacity to the new environment. Since high biomass yield is the primary goal in improving *M. sinensis*, it appears that the correlation between traits and biomass yield during the establishment time may be important in *M. sinensis* breeding programs because plant biomass yield may not be always the optimum criteria for early selection (Gifford et al., 2015). Using traits that can be reliably measured in the early years of establishment to predict future performance could help an efficient early selection to reduce the breeding time. At least, data could be used to remove the unwanted genotypes with little potential.

In this study, plants were not evaluated in the first year after transplanting. In the subsequent 3 years, *M. sinensis* individuals were examined for biomass yield and related morphological traits in Ya'an, southwest of China, an area known as having the richest rainfall but relatively less light for grass species growth. Abundant phenotypic variations of traits in the establishment phase were found in the population. Most of the traits related to biomass yield tended to reach optimum value in the third year after transplanting and were close to stable growing stage. The results were consistent with previous studies, which suggested that a 3 year establishment phase was needed to achieve a stable or reliable population to collect phenotypic data in *Miscanthus* species (Clifton-Brown and Lewandowski, 2000).

Superior genotypes of *M. sinensis* with high tiller numbers and plant height could be comparable to *Miscanthus* × *giganteus* in terms of biomass yield potential (Heaton et al., 2004; Huang et al., 2011). Although field performance was evaluated for only 3 years (a few traits evaluated for the last 2 years) after transplanting in this study, some individuals had comparable or exceeded values relative to *Miscanthus* × *giganteus* in Europe and North America (Lewandowski et al., 2003a,b; Jezowski, 2008; Maughan et al., 2012; Gifford et al., 2015). The results suggested that some *M. sinensis* genotypes with vigorous growth, especially with high tillering capacity, greatly contributed to more biomass yield. Those genotypes would have the genetic potential to match or exceed the biomass yield of *Miscanthus* × *giganteus* in similar climate areas, although the performance of those genotypes has not been tested in colder climates or higher latitudes. In particular, plant height almost reached the optimum at the second year after transplanting and became stable the following year. The results suggested that plant height can be used as early selection criteria to develop genotypes with high biomass yield potential in *M. sinensis*. Thus, it could be possible to develop high biomass yield of *M. sinensis* by simultaneous selecting individuals with high tiller numbers and plant height. Genotypes with high biomass yield identified in this study would be useful for accelerating its domestication as an energy crop in similar areas.

As one of the 34 biodiversity hotspots around the world, southwest China has a special geographical location, climatic conditions, and abundant wild resources (Mittermeier et al., 2000). Prior studies have shown that high gene flow existed among *M. sinensis* populations from southwest China (Xu et al., 2013; Nie et al., 2014), which could be due to an introgression occurred from here to other distribution areas around China (Xiao et al., 2013). By analyzing *trnL-F* and *rpl20-rps12* sequences, Yan et al. (2015) found that the haplotypes “H2” widely distributed among populations from southwest China and had a high level of similarity (99.64%) with haplotypes “A” identified in Japanese *M. sinensis* populations (Shimono et al., 2013). Furthermore, through comparison of haplotypes from NCBI, they determined that haplotypes “H1” and “H6” had relatively high similarity to the haplotypes obtained from the Liaoning and Jilin provinces located in northeast China (Jiang et al., 2013). In this study, the 138 individuals collected from *N 24*°*12*′*15.9*″ to *N 32*°*38*′*57.9*″ across southwest China revealed a very high level of gene flow, which is consistent with previous studies. All the results inferred that *M. sinensis* populations from southwest China have a mixed and complex ancestry owing to the complex ecotypes, random genetic drift, and the high rate of gene flow. Hence, knowing the relationship and population structure of *M. sinensis* from southwest China is important for taxonomic research and phylogenetic evaluation for their conservation and utilization.

Due to the lengthy period of establishment and the challenges in getting phenotypic data from a large population, a marker-assisted selection program would add tremendous value to a *Miscanthus* breeding program. However, different types of markers vary in amplification capacity and relationship to the traits in *Miscanthus* species. SSR regions lie within microsatellite repeats, and have a random distribution genome wide, while the target locus of SRAP is mainly located in open reading frame regions (ORFs). ISAP, as a very good complementary, is designed by using the highly conserved sequence of introns splice position as the core of the primer sequences to amplify the genes encoding areas, which could leading to a high association with expressed sequence. In this study, SRAP have a very high amplification capacity than other markers, demonstrating its values for use in the molecular marker system. The average number of alleles per loci produced in this study was similar to previous studies (Hung et al., 2009; Ho et al., 2011; Zhou et al., 2011; Lu et al., 2012; Nie et al., 2014). Furthermore, both the conserved grass EST-SSRs and ISAP markers were amplified in *M. sinensis* for the first time but proved to be highly efficient markers for *Miscanthus*. Using a large amount of molecular markers has great potential to obtain reliable and important loci for detecting the relationships between markers and traits of interest.

Molecular markers have been used to evaluate the genetic relationship of accessions in *M. sinensis* all around its distributed areas. Some genetic maps with high density and resolution have been constructed (Kim et al., 2012; Ma et al., 2012; Swaminathan et al., 2012; Liu et al., 2015). Atienza's genetic map had been sufficiently used in four QTL studies (Atienza et al., 2003a,b,c,d) in the early stage, but limitations occurred due to the low reproducibility of RAPD markers, the small population size (*N* = 89) and incomplete genetic map (28 linkage groups detected whereas *M. sinensis* has 19 chromosomes). More recently, Gifford et al. (2015) and Liu et al. (2015) conducted QTL studies based on the high density genetic maps (Swaminathan et al., 2012; Liu et al., 2015), but identification of QTLs using the genetic map are still limited. Furthermore, the association studies were lagged than QTL research on Miscanthus, and to date, the only two studies were reported. Zhao et al. (2013) conducted marker-trait association by analyzing a *M. sinensis* population from China and using 23 SSR markers transferable from *Brachypodium distachyon* and 9 markers were significantly (*P* < 0.01) associated with heading date and biomass yield. A genome-wide association study was conducted in a 138 *M. sinensis* population by using 53,174 single-nucleotide variants (SNVs) (Slavov et al., 2014) and a total of 17 significant associations (false discovery rate < 10^−5^) with phenology, morphology, and cell wall composition traits were detected. In our study, 12 significant associations of biomass yield with related traits were identified and marker “793” associated with flag leaf width reached genome-wide significant after Bonferroni correction for multiple testing. The possible reason why we obtained a number of significant associations similar to Slavov et al. (2014) while using a much smaller number of markers could be that the PCR-based markers are more likely to be associated with traits than random SNPs (just based on their distribution in the genome). However, in our study the ability to predict phenotypes seemed lower than that obtained from genome-wide sequencing (Slavov et al., 2014). Other factors like the number of markers and the structure of the population may be equally important in influencing the power of association studies. The phenotypic data and markers result from association study could be potential candidates to supplementing the database of *Miscanthus* for improving genome-wide selection in a breeding program.

## Author contributions

XZ, LH, and GN conceived the project and designed the experiments; GN, XW, and YZ performed the experiments; GN, XY, and XL analyzed the data; XZ, MT, and YJ finalized the manuscript; all authors discussed the results and reviewed the manuscript.

### Conflict of interest statement

The authors declare that the research was conducted in the absence of any commercial or financial relationships that could be construed as a potential conflict of interest.
