# Supplementary material for: Marker-Trait Association for Biomass Yield of Potential Bio-fuel Feedstock Miscanthus sinensis from Southwest China
Source: Front Plant Sci. 2016 Jun 7;7:802. doi: 10.3389/fpls.2016.00802 (PMC4894898; doi:10.3389/fpls.2016.00802)
Supplement: Supplementary file 1 [file DataSheet1.docx]

Supplementary Material

**Marker-Trait Association for Biomass Yield of Potential Bio-fuel Feedstock *Miscanthus sinensis* from Southwest China**

**Gang Nie^1^, Linkai Huang^1^, Xinquan Zhang^1,^*****, Megan Taylor ^2^, Yiwei Jiang^2^, Xinchun Liu^3^, Xinyu Wang^1^, and Yajie Zhang^1^**

*Corresponding author: Xinquan Zhang, E-mail address: [zhangxq@sicau.edu.cn](mailto:zhangxq@sicau.edu.cn)

# Supplementary Figures and Tables

## Supplementary Figures


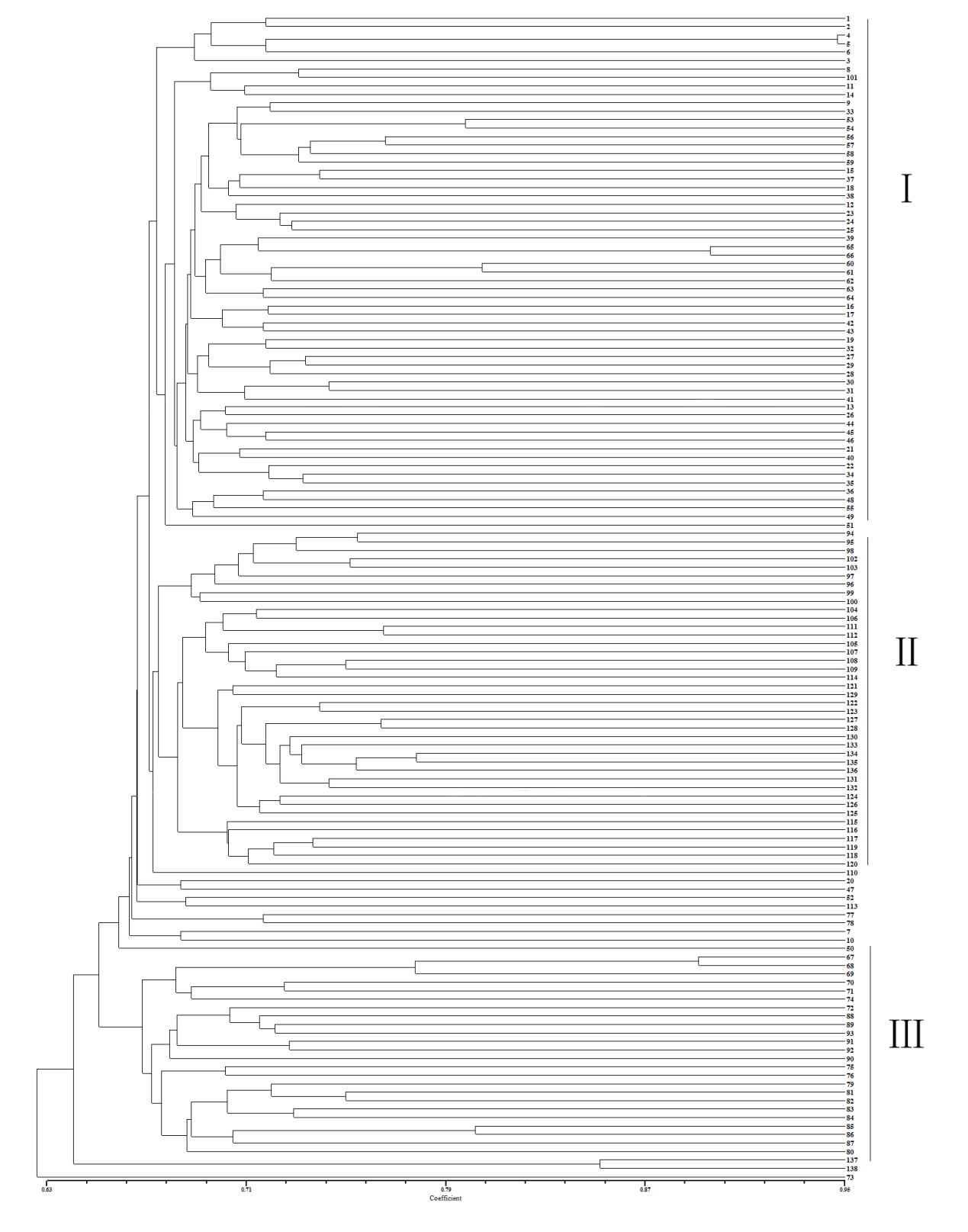


**Supplementary Figure 1** Dendrogram of 138 *M. sinensis* from southwest China based on genetic similarity coefficient.

## Supplementary Tables

**Supplementary Table 1** The primer sequences information in this study.

| Primer Number | Primer  Name | Primer Kind | Primer Sequence F（5'-3'） | Primer Sequence R（5'-3'） |
| --- | --- | --- | --- | --- |
| N-1 | F1+R2 | ISAP | CGATATAAGCAAAGGTAA | CTGCAATGTCCCATAGAT |
| N-2 | F1+R3 | ISAP | CGATATAAGCAAAGGTAA | CTGCAACAAGATCTCAGA |
| N-3 | F1+R8 | ISAP | CGATATAAGCAAAGGTAA | CTGCAATAACCACATGAA |
| N-4 | F2+R2 | ISAP | GCATGAATGCAAAGGTAA | CTGCAATGTCCCATAGAT |
| N-5 | F2+R3 | ISAP | GCATGAATGCAAAGGTAA | CTGCAACAAGATCTCAGA |
| N-6 | F2+R5 | ISAP | GCATGAATGCAAAGGTAA | CTGCAAAATTCAATAGTT |
| N-7 | F4+R3 | ISAP | ACGAAGATGGAAAGGTAA | CTGCAACAAGATCTCAGA |
| N-8 | F5+R1 | ISAP | TAGCCGGTATCAAGGTAA | CTGCAATTAAGCAAGAAC |
| N-9 | F6+R1 | ISAP | CGTCCGATGAAAAGGTAA | CTGCAATGTCCCATAGAT |
| N-10 | F6+R2 | ISAP | CGTCCGATGAAAAGGTAA | CTGCAACAAGATCTCAGA |
| N-11 | F6+R5 | ISAP | CGTCCGATGAAAAGGTAA | CTGCAAAATTCAATAGTT |
| N-12 | F6+R6 | ISAP | CGTCCGATGAAAAGGTAA | CTGCAAATGTTAAACCCA |
| N-13 | F6+R7 | ISAP | CGTCCGATGAAAAGGTAA | CTGCAAGGGTTAACCAGT |
| N-14 | F8+R6 | ISAP | AGCCGTTTATACAGGTAA | CTGCAAATGTTAAACCCA |
| N-15 | F8+R8 | ISAP | AGCCGTTTATACAGGTAA | CTGCAATAACCACATGAA |
| N-16 | F9+R1 | ISAP | CATCTCACTTTCAGGTAA | CTGCAATTAAGCAAGAAC |
| N-17 | F9+R2 | ISAP | CATCTCACTTTCAGGTAA | CTGCAATGTCCCATAGAT |
| N-18 | F9+R5 | ISAP | CATCTCACTTTCAGGTAA | CTGCAAAATTCAATAGTT |
| N-19 | F9+R6 | ISAP | CATCTCACTTTCAGGTAA | CTGCAAATGTTAAACCCA |
| N-20 | F9+R7 | ISAP | CATCTCACTTTCAGGTAA | CTGCAAGGGTTAACCAGT |
| N-21 | M＆Mest2663 | SSR | GTGGGGACTGCTTCAAGTGT | CAGAGTATCGGTCACCACCA |
| N-22 | M＆Mest3185 | SSR | GAATCAATCAAGCCCTCGAC | CGGACACGTTTGCTCTTTG |
| N-23 | M＆Mest3642 | SSR | CTAGCTTGCTTGACGCCATA | GATACGGTGTTCATGGCTGA |
| N-24 | M＆Mest3665 | SSR | CACAAAGCTTCTCTCCTCGAT | GGCTTCTTCTGCCAGTCGT |
| N-25 | M＆Mest6656 | SSR | GGGAGAGAATTTGTTAGGTCTCC | CGCTCCTCTGATCTTGATCTT |
| N-26 | M＆Mest7737 | SSR | TCAGGTCGGTTAGTCGCTTC | GTGTTCCGGTGTGTGTTTTG |
| N-27 | M＆Mest9817 | SSR | AGCACTTGTCCACCACGTC | CTTCTTGTCGTCGCTGTCTG |
| N-28 | M＆Mest12682 | SSR | GTCAGGTCTCCGTCCTCATC | ATCCGACTACCGCCCAAT |
| N-29 | M＆Mest12817 | SSR | AGGGGTAACGAGGCATTCA | GCAACATCGTTGAAACCTGA |
| N-30 | M＆Mest15736 | SSR | GAAGCACGTACGCAACCCTA | CAAATGTAGCATCCGTGGTG |
| N-31 | M＆M21 | SSR | GCATATATTGACCTATGTGTG | TAGCTTCGTTCCATCTCCAT |
| N-32 | M＆M29 | SSR | CAAGTCTCTAAAGTCAATGTG | CCCGGTCTTTTCCGTTAATT |
| N-33 | M＆M39 | SSR | AGAAATGAAAGTGCAGTGACA | AAGGAGTGCTTCTCCCTCTC |
| N-34 | MSSR4 | SSR | TTCTGTGAGATTCTGGTATGCC | CAACTTGCTTGGGACTGA |
| N-35 | MSSR11 | SSR | TTGAAGAGGGTAGCGGTTG | TAGTTAGGGGCTGTTTGGA |
| N-36 | MSSR14 | SSR | ACTAAAGGCGAAAGCTAGGAGG | CAGATGCTGGCTGTTGGTGATGT |
| N-37 | MSSR17 | SSR | CTATGATGATGGCAACG | TCCAAAACAGTGAGGGT |
| N-38 | MSSR18 | SSR | TTTTCTGCCCACTACTGCTA | TGTGATCTTCTATGCTTCCA |
| N-39 | MSSR21 | SSR | TATGGGTGAATGTTGGTTT | GCCCGTTTGTGCGAGTGC |
| N-40 | MSSR25 | SSR | TGACAGGCACAGAAAGC | CCAACCATCAAGCAGGAG |
| N-41 | MSSR30 | SSR | GACCTTTCAGCCACCCTC | AACGACTCCTGCTCCTATCA |
| N-42 | MSSR36 | SSR | TAAGCCCAAACAAAGGAAAT | CAAATGGCAATAGTGAGCAA |
| N-43 | MSSR37 | SSR | CAGATGCCATTACTGTAGCGA | ACCACAACGAAACCAAAAC |
| N-44 | MSSR42 | SSR | TGCCACGCCTTCTTCACCTATC | GCATCCAGCCATCCACCCTC |
| N-45 | MSSR70 | SSR | GCACGCATGAGCCAAACTG | TCGGTCGGTGCTTGTCTCG |
| N-46 | HAU-12 | SSR | CACCAACGCCAATTAGCATCC | GTGGGCGTGTTCTCCTACTACTCA |
| N-47 | HAU-58 | SSR | TAAAGCTATGATGGCACTTGCAGA | CATATTTGCCTTTGCCCTTTTGTA |
| N-48 | HAU-101 | SSR | ACCCCCTGATTCTCTCTTACGTTT | CTGGATGAGGAGGAAGAATACGAG |
| N-49 | HAU-130 | SSR | GGACAGCTTGGCTTCGAGTG | ACGTTGGCCGTTAGTTCTTATCCT |
| N-50 | HAU-170 | SSR | ACAGAAACCAATGCATGTGATGAG | TGCATGGTTGCTTCAGCAGT |
| N-51 | HAU-187 | SSR | CCAGACATTCCCCAAACCCTA | CGTCGGTGTCGTACTGGTTG |
| N-52 | HAU-196 | SSR | TCAATCAAGCCTCTCGTAAGGAAC | CTCTTGATCTCAACCGAAATCCTG |
| N-53 | HAU-205 | SSR | ACCGTCTCAGCAAAATGGTC | CCGCCTTCACTATGGTCAAT |
| N-54 | HAU-372 | SSR | GTAGAGATCGATTCGCTAACCTGC | AGTTGTTCCGTTCCGTCCTTATC |
| N-55 | HAU-384 | SSR | ACAGGACTGCAGTGTCAGGAT | GGGTTTCTCAAACTCCTTTGG |
| N-56 | HAU-414 | SSR | AACTAATTTGCATGGCAGCAT | GCGGACAAGCAAGTAGATGTG |
| N-57 | mSSCIR2 | SSR | ATGCACGTCCTTCCGCCTT | GATTCTTTTGAGCTCCCAGTTG |
| N-58 | mSSCIR10 | SSR | ACACCACTCACATCCACTTG | TGATACACCATTGTTGATGC |
| N-59 | mSSCIR34 | SSR | ATCGCCTCCACTAAATAAT | TTGTCTTTGCTTCCTCCTC |
| N-60 | mSSCIR43 | SSR | ATTCAACGATTTTCACGAG | AACCTAGCAATTTACAAGAG |
| N-61 | SMC31CUQ | SSR | CATGCCAACTTCCAATACAGACT | AGTGCCAATCCATCTCAGAGA |
| N-62 | SEGMS840 | SSR | TCGGATTCTCTGATTGTATGTA | ACTATTGATGAACTAACCGTCC |
| N-63 | SMC640CS | SSR | GCGATGGTTCCTATGCAACTT | TTCGTGGCTGAGATTCACACTA |
| N-64 | SG1 | SSR | ATTCTGACTTAACCCACCCCTAAA | AGCTCATCAATGTCCCAAACC |
| N-65 | SG2 | SSR | GGGCAATCTTGATGGCGACAT | AGGTGTGGCTCGGGGAGAAC |
| N-66 | SG9 | SSR | TCTGGCCATGACTTATCAC | AAATGGCGTAGACTCCCTTG |
| N-67 | SG11 | SSR | CAGCAACTTGCACTTGTC | GGGAGCAATTTGGCACTAG |
| N-68 | SG16 | SSR | GCAAGCGAGCTGACTTATGTAACGAGA | CAAAGTGCTACTAAACCTATGCAGGGTGAA |
| N-69 | SG24 | SSR | ATCGGATCCGTCAGATC | TCTAGGGAGGTTGCCAT |
| N-70 | SG26 | SSR | TGGCGGACATCCTATT | GGAGAGCCCGTCACTT |
| N-71 | cnl 39 | SSR | TACCTGTGCGGCGATGAAT | CAGGAGCAGGAGAACGTGAA |
| N-72 | cnl 51 | SSR | CTAGGGTTTCCCACCTCTCA | AATGTCCTTGGCGTTGCT |
| N-73 | cnl 53 | SSR | CGCAGCAAGTAGGGTTAGGA | CCTCGTGGTGGATCTGCAT |
| N-74 | cnl 55 | SSR | GCTGATAGCGAGGTGGGTAG | CTGCCGGTTGATCTTGTTCT |
| N-75 | cnl 74 | SSR | GGCTCGAGCTTAAAACCCTA | CTCCATCCATTCTTGCCATCT |
| N-76 | cnl 130 | SSR | AAATGTTGAGCAACGGGAGCT | ACTTCATAGGGCGGAGGTCT |
| N-77 | cnl 144 | SSR | AGAAGGCGGCTCAGAAGAAG | GCTCCAACTCAGAATCAACAA |
| N-78 | cnl 147 | SSR | GGCTAGGGTTTCGACTCCTC | AGATGGCGAACTCGACCTG |
| N-79 | cnl 152 | SSR | ACAAAGGCTCACCGTGGAA | GTCGGAGGCGATGAACTCT |
| N-80 | cnl 156 | SSR | CTTCCCTCACCTCAAGCAGT | GACAGCAGCACACCGAGTT |
| N-81 | Me1+em3 | SRAP | TGAGTCCAAACCGGATA | GACTGCGTACGAATTGAC |
| N-82 | Me2+em1 | SRAP | GACTGCGTACGAATTTGC | GACTGCGTACGAATTAAT |
| N-83 | Me3+em5 | SRAP | TGAGTCCAAACCGGAAT | GACTGCGTACGAATTACC |
| N-84 | Me5+em2 | SRAP | TGAGTCCAAACCGGAAG | GACTGCGTACGAATTTGC |
| N-85 | Me6+em7 | SRAP | TGAGTCCAAACCGGTAA | GACTGCGTACGAATTCAA |
| N-86 | Me7+em1 | SRAP | TGAGTCCAAACCGGTCC | GACTGCGTACGAATTAAT |
| N-87 | Me8+em5 | SRAP | TGAGTCCAAACCGGTGC | GACTGCGTACGAATTACC |
| N-88 | Me10+em1 | SRAP | TGAGTCCAAACCGGTTG | GACTGCGTACGAATTAAT |
| N-89 | Me1+em8 | SRAP | TGAGTCCAAACCGGATA | GACTGCGTACGAATTCTG |
| N-90 | Me3+em9 | SRAP | TGAGTCCAAACCGGAAT | GACTGCGTACGAATTCGA |
| N-91 | Me5+em4 | SRAP | TGAGTCCAAACCGGAAG | GACTGCGTACGAATTTGA |
| N-92 | Me6+em8 | SRAP | TGAGTCCAAACCGGTAA | GACTGCGTACGAATTCTG |
| N-93 | Me7+em10 | SRAP | TGAGTCCAAACCGGTCC | GACTGCGTACGAATTCAG |
| N-94 | Me9+em1 | SRAP | TGAGTCCAAACCGGTAG | GACTGCGTACGAATTAAT |
| N-95 | Me10+em2 | SRAP | TGAGTCCAAACCGGTTG | GACTGCGTACGAATTTGC |
| N-96 | Me3+em10 | SRAP | TGAGTCCAAACCGGAAT | GACTGCGTACGAATTCAG |
| N-97 | Me5+em8 | SRAP | TGAGTCCAAACCGGAAG | GACTGCGTACGAATTCTG |
| N-98 | Me6+em10 | SRAP | TGAGTCCAAACCGGTAA | GACTGCGTACGAATTCAG |
| N-99 | Me7+em5 | SRAP | TGAGTCCAAACCGGTCC | GACTGCGTACGAATTACC |
| N-100 | Me8+em7 | SRAP | TGAGTCCAAACCGGTGC | GACTGCGTACGAATTCAA |
| N-101 | Me9+em9 | SRAP | TGAGTCCAAACCGGTAG | GACTGCGTACGAATTCGA |
| N-102 | Me4+em7 | SRAP | TGAGTCCAAACCGGACC | GACTGCGTACGAATTCAA |
| N-103 | Me5+em10 | SRAP | TGAGTCCAAACCGGAAG | GACTGCGTACGAATTCAG |
| N-104 | Me9+em8 | SRAP | TGAGTCCAAACCGGTAG | GACTGCGTACGAATTCTG |
